# miR-203 and miR-320 Regulate Bone Morphogenetic Protein-2-Induced Osteoblast Differentiation by Targeting Distal-Less Homeobox 5 (*Dlx5*)

**DOI:** 10.3390/genes8010004

**Published:** 2016-12-23

**Authors:** Navya Laxman, Hans Mallmin, Olle Nilsson, Andreas Kindmark

**Affiliations:** 1 Department of Medical Sciences, Uppsala University, Uppsala 75185, Sweden; andreas.kindmark@medsci.uu.se; 2 Science for Life Laboratory, Department of Medical Sciences, Uppsala University Hospital, Uppsala 75185, Sweden; 3 Science for Life Laboratory, Department of Biochemistry and Biophysics, Stockholm University, Stockholm 17121, Sweden; 4 Department of Surgical Sciences, Uppsala University, Uppsala 75185, Sweden; Hans.Mallmin@akademiska.se (H.M.); Olof.Nilsson@akademiska.se (O.N.)

**Keywords:** miRNA, *Dlx5*, BMP-2, osteoblast differentiation

## Abstract

MicroRNAs (miRNAs) are a family of small, non-coding RNAs (17–24 nucleotides), which regulate gene expression either by the degradation of the target mRNAs or inhibiting the translation of genes. Recent studies have indicated that miRNA plays an important role in regulating osteoblast differentiation. In this study, we identified miR-203 and miR-320b as important miRNAs modulating osteoblast differentiation. We identified *Dlx5* as potential common target by prediction algorithms and confirmed this by knock-down and over expression of the miRNAs and assessing *Dlx5* at mRNA and protein levels and specificity was verified by luciferase reporter assays. We examined the effect of miR-203 and miR-320b on osteoblast differentiation by transfecting with pre- and anti-miRs. Over-expression of miR-203 and miR-320b inhibited osteoblast differentiation, whereas inhibition of miR-203 and miR-320b stimulated alkaline phosphatase activity and matrix mineralization. We show that miR-203 and miR-320b negatively regulate BMP-2-induced osteoblast differentiation by suppressing *Dlx5*, which in turn suppresses the downstream osteogenic master transcription factor *Runx2* and *Osx* and together they suppress osteoblast differentiation. Taken together, we propose a role for miR-203 and miR-320b in modulating bone metabolism.

## 1. Introduction

Bone Morphogenetic Proteins (BMPs) are powerful cytokines that are capable of inducing ectopic bone formation [1]. BMPs are a part of the transforming growth factor-β (TGFβ) superfamily [2] and they play a role in bone and cartilage formation by inducing the differentiation of mesenchymal progenitors into the osteoblast lineage. BMPs stimulate osteoblast differentiation through the modulation of BMP receptors and signal transducers Smad1 and Smad5 [3,4,5] and osteoblast-specific transcription factor Distal-less Homeobox 5 (*Dlx5*) [6,7], Runt-related transcription factor 2 (*Runx2*) [8,9], and Osterix (*Osx*) [10].

The expression of the bone-inducing transcription factor, *Dlx5*, is associated with osteoblast differentiation [11] and exhibits its highest expression when extracellular matrix mineralizes at the final stage of osteoblast differentiation. Studies show that in BMP-2-induced osteoblast differentiation, *Dlx5* plays an essential role in upregulation of the downstream osteogenic master transcription factor *Runx2* and *Osx*, whose expression in turn sequentially regulates the expression of osteoblast specific genes to induce osteoblast differentiation [8,12]. Concomitantly, *Dlx5* inhibits adipogenic differentiation by inhibiting *PPARγ* (Peroxisome proliferator-activated receptor gamma) expression in bone marrow mesenchymal stem cells (MSCs) [13].

*Runx2* is the master transcription factor for osteoblast differentiation. *Runx2* expression is substantially upregulated by BMP2 through the activation of Smad signaling [9,14]. The physical interaction of *Runx2* with *Smad1* and *Smad5* is necessary to enhance its transcriptional and osteogenic activity. Studies have shown that the BMP2-Smad-Runx2 axis is important for osteoblast differentiation. This complex also induces alkaline phosphatase (ALP) activity and the expression of other osteoblast-specific genes.

Osterix is a novel zinc finger-containing transcription factor, which acts downstream of Runx2, and its expression is essential for osteoblast differentiation. During the differentiation of mesenchymal cells into osteoblasts, the expression of Osterix is considerably upregulated by BMP-2, suggesting the BMP2 acts upstream of Osterix during osteoblast differentiation [10].

MicroRNAs (miRNAs) are an abundant class of small, single-stranded non-coding RNAs (~22 nucleotides long). They have emerged as important post-transcriptional regulators in diverse processes, e.g., cell proliferation and differentiation [15,16]. MiRNAs anneal to the 3′ UTR of their target genes and regulate protein translation and/or mRNA stability [17]. There is an increasing number of miRNAs identified recently that contribute to the regulation of osteoblast differentiation and bone formation. MiRNAs may target negative regulators of osteogenesis and as a result operate as positive regulators, or they may act as negative regulators by targeting important osteogenic factors. As a result, miRNAs exert control over skeletal gene expression [18,19,20,21]. Studies show that several bone-inducing transcription factors and signaling molecules that are involved in the function and differentiation of MSCs to osteoblasts are targets of miRNAs. Recent studies reported many bone-regulating miRNAs (“osteomiRs”) that orchestrate BMP2-induced osteogenesis, such as miR-141 and miR-200a by downregulating *Dlx5* represses BMP2-induced pre-osteoblast differentiation [22]. miR-133 directly targets *Runx2*, and miR-135 attenuates Smad5 pathway and inhibits osteoblast differentiation [23]. Studies also show that mir-93 attenuates osteoblast mineralization by directly targeting *Osx* [24], whereas mir-206 targets connexin 43 and inhibits osteogenesis [25].

In the present study, we aimed to identify miRNAs involved in BMP2-induced osteogenesis using primary human osteoblasts (HOBs). Our results show that miR-203 and miR-320b target *Dlx5*, a bone-inducing transcription factor, and together they suppress osteoblast differentiation. These results were confirmed by Western blot analysis, real-time PCR, and luciferase reporter assays. The activity of miR-203 and miR-320b were modulated using an antimiR oligonucleotide, which markedly increased osteogenic differentiation in vitro, whereas miR-203 and miR-320b overexpression reversed these effects. This study confirmed earlier reports that *Dlx5* is a common upstream regulator of *Runx2* and *Osx*, and both genes are regulated independently by *Dlx5*. These results strongly suggest that miR-203 and miR-320b suppresses BMP-induced osteogenic differentiation by suppressing *Dlx5* and its downstream signaling.

## 2. Materials and Methods

### 2.1. Bone Cell Culture

Primary human osteoblast (HOB) cells were isolated from human trabecular bone collected from 3 donors undergoing total hip replacement as published previously [26,27,28]. The bone chips were washed thoroughly and minced with PBS. The minced bone chips were cultured in medium containing α-MEM (Sigma-Aldrich, Haverhill, UK) supplemented with 2 mmol/L l-glutamine, 100 U/mL penicillin, 100 mg/mL streptomycin and 10% fetal bovine serum (Sigma-Aldrich) at 37 °C with 5% CO_2_ until confluence was reached. The culture medium was changed twice weekly. The study was approved by the local ethics committee (Ethical approval # Ups 03-561).

Cells from second passage were trypsinized and seeded at a density of 35,000 cells/well in 24 well plates and grown to 60%–80% confluency for transfection. miR-203 and miR-320 were over expressed and inhibited by transfecting the cells with mirVana hsa-miR-203 mimic (Ambion, Catalog No. 4464066), mirVana hsa-miR-203 inhibitor (Ambion, Catalog No. 4464084), mirVana hsa-miR-320 mimic (Ambion, Catalog No. 4464066), mirVana hsa-miR-320 inhibitor (Ambion, Catalog No. 4464084) using mirVana miRNA inhibitor negative control #1 (Ambion, Catalog No. 4464077) and mirVana miRNA mimic negative control #1 (Ambion, Catalog No. 4464061) at 40 nM concentrations with Magnet Assisted Transfection (MATra-si) reagent (IBA GmbH, Göttingen, Germany) according to the manufacturers′ protocols. Each transfection was performed in triplicate. The cells were incubated for 24 h and stimulated with 300ng/mL of recombinant human BMP-2 (InductOs, Pfizer, New York, NY, US) and control cells were left untreated. Cells were harvested at the over a period of 7 days at intervals of 2 h, 12 h, 1 day, 2 days, 3 days, 5 days and 7 days.

### 2.2. Total RNA Extraction

The HOB cells were harvested at the seven different time points. The cell lysates were homogenized using QIAshredder (Qiagen, Hilden, Germany). RNA was extracted from the cell lysates using the RNeasy Mini Kit (Qiagen). Agilent 2100 BioAnalyzer (Agilent Technologies, Palo Alto, CA, USA) was used to confirm high RNA quality for all samples, RIN values in our study were between 8.2 and 9.5. The concentrations were determined with NanoDrop ND-1000 (NanoDrop Technologies, Wilmington, DE, USA) with an OD 260/280 between 1.95 and 2.03.

### 2.3. Target Prediction

Target prediction tools used to predict the targets of differentially expressed miRNA in this study were, e.g., TargetScan, Release 6.2: June 2012 (http://www.targetscan.org/), PicTar (http://pictar.mdc-berlin.de/) [29], and the miRanda algorithm [30,31].

### 2.4. Quantification by Real-Time PCR

Twenty nanograms of total RNA from each time point was reverse transcribed using TaqMan MicroRNA Reverse Transcription Kit (Applied Biosystems, Foster City, CA, USA), according to the manufacturer’s instructions, enabling miRNA specific cDNA synthesis for the miR-203 and miR-320 human miRNAs and 1 miRNA control.

Following the RT step, TaqMan MicroRNA Assays (Applied Biosystems) were performed using specific TaqMan miRNA probes hsa-miR-320b (ID 002844) and hsa-miR-203 (ID 000507) (Applied Biosystems) according to the manufacturer’s instructions. PCR cycling began with AmpliTaq Gold enzyme activation at 95 °C for 10 min, then 40 cycles of 95 °C for 15 s, and 60 °C for 60 s performed on a 7500 Fast Real-Time PCR System (Applied Biosystems).

For the target osteogenic genes cDNA synthesis was performed in triplicate using total RNA reverse transcribed using High Capacity cDNA reverse transcription kit (Applied Biosystems), according to the manufacturer′s instructions, with no template control added to ensure a lack of signal in assay background. The real-time PCR reactions were carried out with 10 µL of 2x TaqMan^®^ Universal PCR Master Mix, no AmpErase^®^ UNG (Applied Biosystems), 9 µL diluted cDNA, and 1 µL of TaqMan gene specific assay mix in a 20 µL final reaction volume. Reference gene beta-actin (*ACTB*) and *GAPDH* (Applied Biosystems) was selected as control for normalization of TaqMan data. Probes specific for *Runx2* (Hs00231692_m1), *Dlx5* (Hs00193291_m1), *Osx* (Hs01866874_s1), *ACTB* (Hs01060665_g1) and *GAPDH* (Hs02758991_g1) were purchased from Applied Biosystems. The amplification was carried out using the 7500 Fast Real-Time PCR System (Applied Biosystems) using a 40-cycle program. The 7500 software automatically calculates raw Ct (cycle threshold) values.

The comparative quantitation 2^−ΔΔCT^ method (also called the ΔΔCT method) [32] was used to compare differences in cycle number thresholds for samples normalized for endogenous controls.

### 2.5. Western Blot Analysis

The total cell lysates were prepared at seven different time points using RIPA lysis buffer (50 mM Tris-HCl, pH 8.0, with 150 mM NaCl, 1.0% Igepal CA-630 (NP-40), 0.5% sodiumdeoxycholate, 0.1% SDS, supplemented with 1.0% protease inhibitor cocktail (SIGMA-ALDRICH^®^, St. Louis, MO, USA). Lysates were centrifuged at 10,000-RPM for 20 min to collect supernatant. Coomassie Plus—The Better Bradford Assay^TM^ Reagent (Thermo Scientific, Waltham, MA, USA) was used to quantify the proteins. Twenty micrograms of soluble protein was subjected to SDS-PAGE and the separated proteins were transferred to polyvinylidene difluoride membrane (Millipore). The primary antibodies were used to probe the protein bands against Dlx5 (1:500 dilution; SIGMA-ALDRICH^®^), Runx2 (1:250 dilution, SIGMA-ALDRICH^®^), Osx (1:500 dilution; SIGMA-ALDRICH^®^) and ACTB (1:1000 dilution; Cell Signaling Technology^®^, Danvers, MA, USA). Anti-Rabbit-HRP conjugated secondary antibodies (1:3000 dilution; R&D Systems^®^, Minneapolis, MN, USA) were used to detect the primary antibodies, followed by the target protein visualization with EMD Millipore Immobilon™ Western Chemiluminescent HRP Substrate (ECL). Images were acquired using LI-COR Odyssey^®^ Fc Dual-Mode Imaging system (LI-COR^®^ Biosciences, Lincoln, NE, USA) and Image Studio Software (LI-COR^®^ Biosciences).

### 2.6. Luciferase Reporter Assay

After in silico target prediction as described above, the target region for miR-203 and miR-320 in *Dlx5* was verified using Dual-Luciferase Reporter Assay. The psiCHECK-2 vector, a dual-luciferase plasmid, has both the synthetic Firefly Luciferase (Fluc) gene and the synthetic *Renilla Luciferase* (*hRluc*) gene incorporated, each possessing its own promoter and poly (A)-addition sites. Luciferase reporter plasmids were constructed by inserting a perfectly complementary (Wild type) 3′ UTR fragment of *Dlx5* between the XhoI–NotI restriction sites in the multiple cloning regions in the hRluc gene in the psiCHECK-2 vector (Promega, Madison, WI, USA) by Generay Company (Shanghai, China). We also constructed 3 mutant types by replacing 6–8 base pairs at the 3′-UTR of the seed sequence. DNA sequencing was employed confirming the nucleotide sequences of the constructed plasmids.

*Luciferase* assays were conducted in 96 well plates cells, were HOBs were co-transfected with 100 ng/well of the wild reporter plasmid or the 3 mutant reporter plasmid and 40 nM of mirVana hsa-miR-203 mimic (Ambion, Catalog No. 4464066), mirVana hsa-miR-203 inhibitor (Ambion, Catalog No. 4464084), mirVana hsa-miR-320b mimic (Ambion, Catalog No. 4464066), mirVana hsa-miR-320b inhibitor (Ambion, Catalog No. 4464084) or with mirVana miRNA inhibitor negative control #1 (Ambion, Catalog No. 4464077) and mirVana miRNA mimic negative control #1 (Ambion, Catalog No. 4464061) negative control (NC) using 0.2 μL/well DharmaFECT™ Duo, Dharmacon™. Forty-eight hours post transfection, cells were lysed using passive lysis buffer. *Firefly* and *Renilla luciferase* activity were measured consecutively using the Dual-Luciferase^®^ Reporter Assay System (Promega, Cat. #E1910) by using Lumat LB 9507 luminometer (Berthold Technologies) per manufacturer′s instructions. *Renilla luciferase* activity was normalized to that of *Firefly luciferase*. All experiments were performed in triplicates. The relative luciferase activity was expressed as a ratio to the negative control miRNA.

### 2.7. Statistical Analysis

The statistical difference between pairs of groups was determined by student′s t-test. Two-way analysis of variance (ANOVA) was used to evaluate the statistical significance for comparisons within groups. *p* < 0.05 was considered as statistically significant. Data were analyzed using Statistica v12 software (Stat Soft Inc., Tulsa, OK, USA,) and GraphPad Prism software package (version 6.0, GraphPad Software, Inc., La Jolla, CA, USA).

## 3. Results

### 3.1. miR-203 and miR-320b Targets Dlx5 as Shown by in Silico Analyses

**Figure 1:**
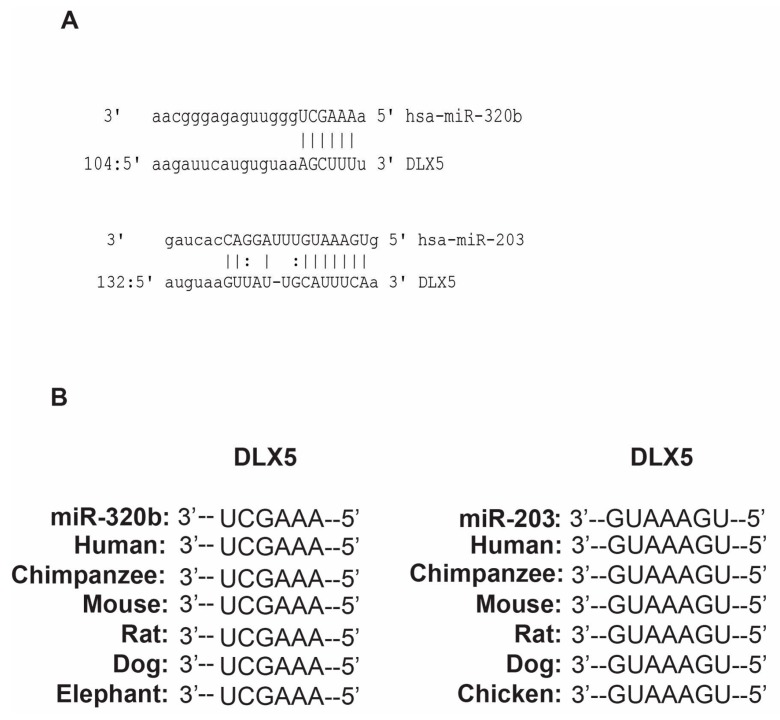
Putative binding sites and interspecies conservation: (**A**) the putative binding sites for miR-320b and miR-203 in the *Dlx5* 3′ UTR as predicted by TargetScan, PicTar and miRanda; and (**B**) seed sequences for miR-320b and miR-203 show conservation between species as depicted in the TargetScan alignment.

In a BMP-2 induced osteoblast differentiation model, we aimed to determine target genes for miR-203 and miR-320b, that we previously had identified as miRNAs important in modulating osteoblast differentiation [33]. The putative binding sites for miR-203 and miR-320b were predicted using TargetScan, PicTar and miRanda, and *Dlx5* was selected as one of the candidate target genes (Figure 1A). The seed sequences of these miRNAs were conserved across species (Figure 1B).

### 3.2. BMP-2 Stimulates Dlx5, Runx2 and Osx Expression

**Figure 2:**
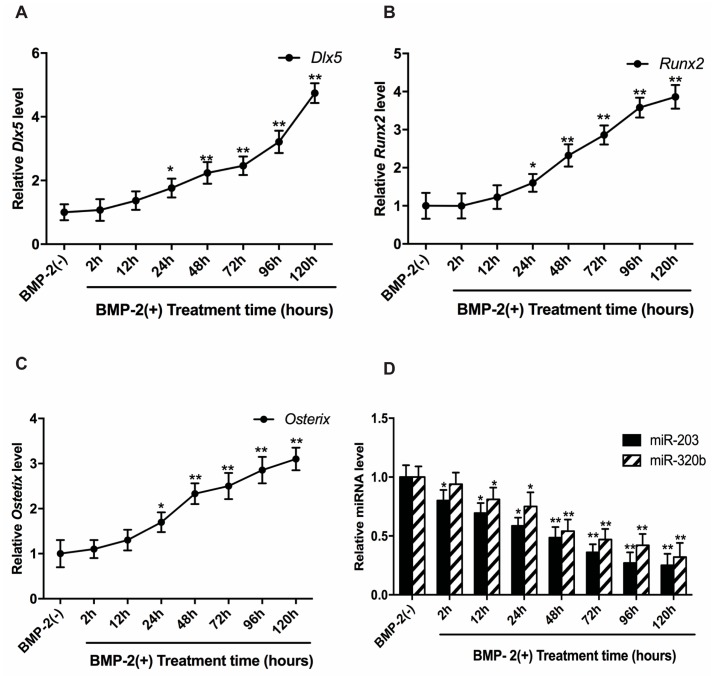
Time course for the expression of *Dlx5*, *Runx2*, *Osx* and miR-203 and miR-320b. The time course shows an increase in the relative expression levels of: *Dlx5* (**A**); *Runx2* (**B**); and *Osx* (**C**) at the mRNA level in BMP-2 induced cells. A concomitant decrease in the miR-203 and miR-320b expression levels is seen in the cells treated with BMP-2 (**D**). Expression levels in cells untreated with BMP-2 were set to 1. All values are represented as mean ± S.D. of three independent experiments. Statistical significance is indicated as * *p* < 0.05 and ** *p* < 0.001.

We examined the expression pattern of *Dlx5*, *Runx2* and *Osx* in human osteoblast cells when stimulated with BMP-2 at different time intervals. In the absence of BMP-2, the expressions of these genes are low, but under BMP-2 stimulation, the HOBs displayed a gradual increase in expression of *Dlx5*, *Runx2* and *Osx* mRNA up to 120 h (Figure 2A–C). The expression of miR-203 and miR-320b in BMP-2 stimulated cells showed a significant down regulation up to 120 h compared to cells untreated cells (Figure 2D).

### 3.3. miR-203 and miR-320b Regulate the Expression of Osteogenic Transcription Factor Dlx5

**Figure 3:**
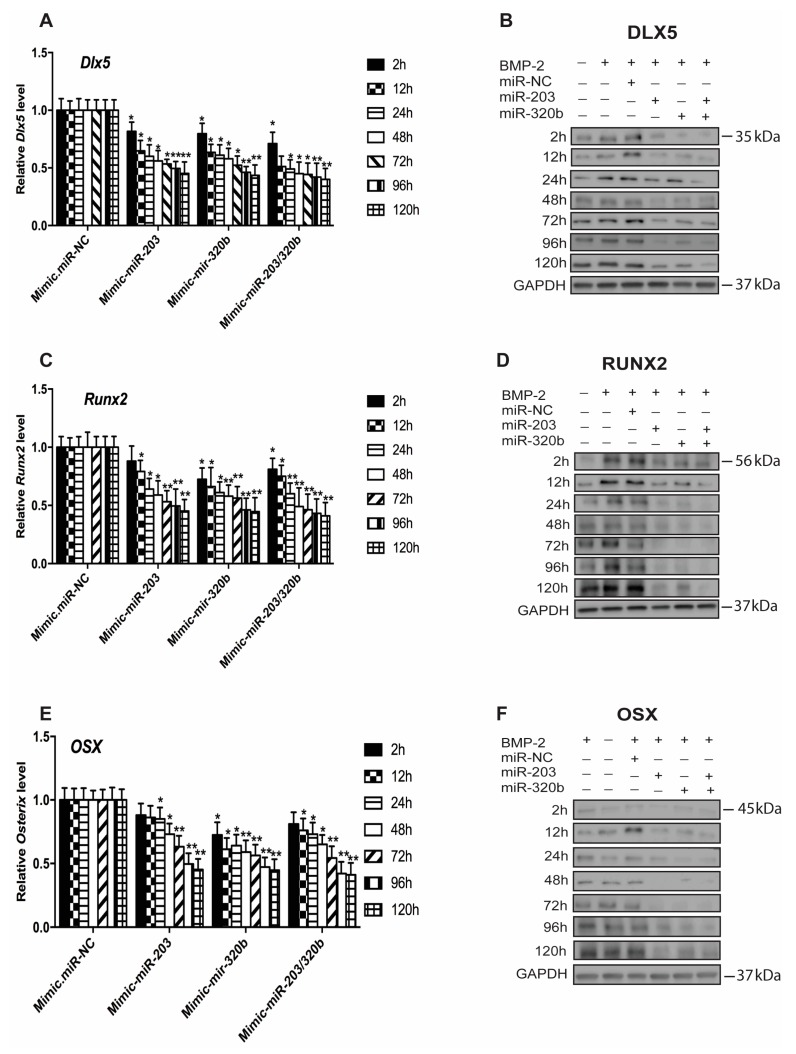
Overexpression of miR-203 and miR-320b negatively regulates *Dlx5*, *Runx2* and *Osx* in osteoblast differentiation. Primary human bone cells were transfected with mimic-miRNA-203 and mimic-miR-320b and RNA and protein isolated at different time points. q-PCR analysis of: *Dlx5* (**A**); *Runx2* (**C**); and *Osx* (**E**) showed down regulation when compared to the mimic-miRNA negative control. mRNA levels were normalized to *GAPDH*. Down regulation at the protein levels of: (**B**) Dlx5, Lane 1, BMP-2 (−); lane 2, BMP-2 (+); (**D**) Runx2, Lane 1, BMP-2 (–); lane 2, BMP-2 (+); and (**F**) Osx, Lane 1, BMP-2 (+); lane 2, BMP-2 (−); lane 3, BMP-2 (+) and mimic-miRNA negative control; lane 4, BMP-2 (+) and miR-203; lane 5, BMP-2 (+) and miR-320b; lane 6, BMP-2 (+) and miR-203 and miR-320b; with GAPDH as a loading control. All values are represented as mean ± S.D. of three independent experiments. Statistical significance is indicated as * *p* < 0.05 and ** *p* < 0.001.

To verify experimentally if the putative binding sites of miR-203 and miR-320b were functional and to evaluate the role of miR-203 and miR-320b on osteoblast differentiation stimulated by BMP-2, HOBs were transfected with miR-203 and miR-320b mimic, anti-miR and NC. mRNA and protein levels were assessed at set time points using qPCR and Western blotting. miRNAs were overexpressed by transfecting HOBs with either miR-203 or miR-320b mimics or both, showing that endogenously expressed miRNA significantly reduced the expression of *Dlx5* (Figure 3A,B). A concurrent decrease in the levels of *Runx2* (Figure 3C,D) and *Osx* (Figure 3E,F) at the mRNA and protein levels up to 120 h when compared to cells treated with mimic NC was also observed.

**Figure 4:**
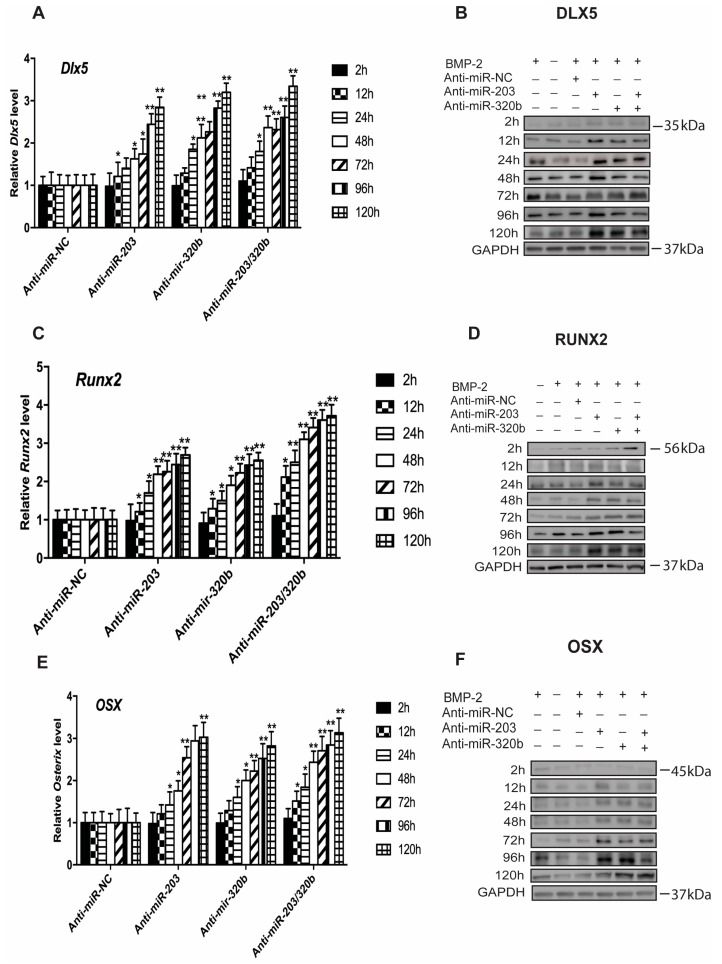
Knockdown of miR-203 and miR-320b up regulates *Dlx5*, *Runx2* and *Osx* in osteoblast differentiation. Primary human bone cells were transfected with pre-miRNA-203 and pre-miR-320b and RNA and protein isolated at different time points. q-PCR analysis of: *Dlx5* (**A**); *Runx2* (**C**); and *Osx* (**E**) showed up regulation when compared to the pre-miRNA negative control. mRNA levels were normalized to GAPDH. Up regulation at the protein levels of: (**B**) Dlx5, Lane 1, BMP-2 (+); lane 2, BMP-2 (−); (**D**) Runx2, Lane 1, BMP-2 (−); lane 2, BMP-2 (+); and (**F**) Osx, Lane 1, BMP-2 (+); lane 2, BMP-2 (−); lane 3, BMP-2 (+) and anti-miRNA negative control; lane 4, BMP-2 (+) and anti-miR-203; lane 5, BMP-2 (+) and anti-miR-320b; lane 6, BMP-2 (+) and anti-miR-203 and -320b; with GAPDH as a loading control. All values are represented as mean ± S.D. of three independent experiments. Statistical significance is indicated as * *p* < 0.05 and ** *p* < 0.001.

Conversely, the expression of either miR-203 or miR-320b or both was repressed using anti-miR, to assess if miRNA repression regulated osteoblast differentiation. *Dlx5* expression was significantly increased (Figure 4A,B), and also observed was a concurrent increase in the levels of Runx2 (Figure 4C,D) and Osx (Figure 4E,F) at the mRNA and protein levels up to 120 h when compared to cells treated with anti-miR NC. GAPDH was used as an internal control for the Western blots. GAPDH for only one time point (120 h) is shown as the representative blot for all the time points. GAPDH levels for the rest of the time points are similar. All these results suggest that miR-203 and miR-320b negatively regulates *Dlx5* expression at translational level in BMP-2 stimulated osteoblast differentiation, and in turn regulates *Runx2* and *Osx* expression.

### 3.4. miR-203 and miR-320b Regulates BMP-2 Stimulated Human Osteoblast Differentiation

**Figure 5:**
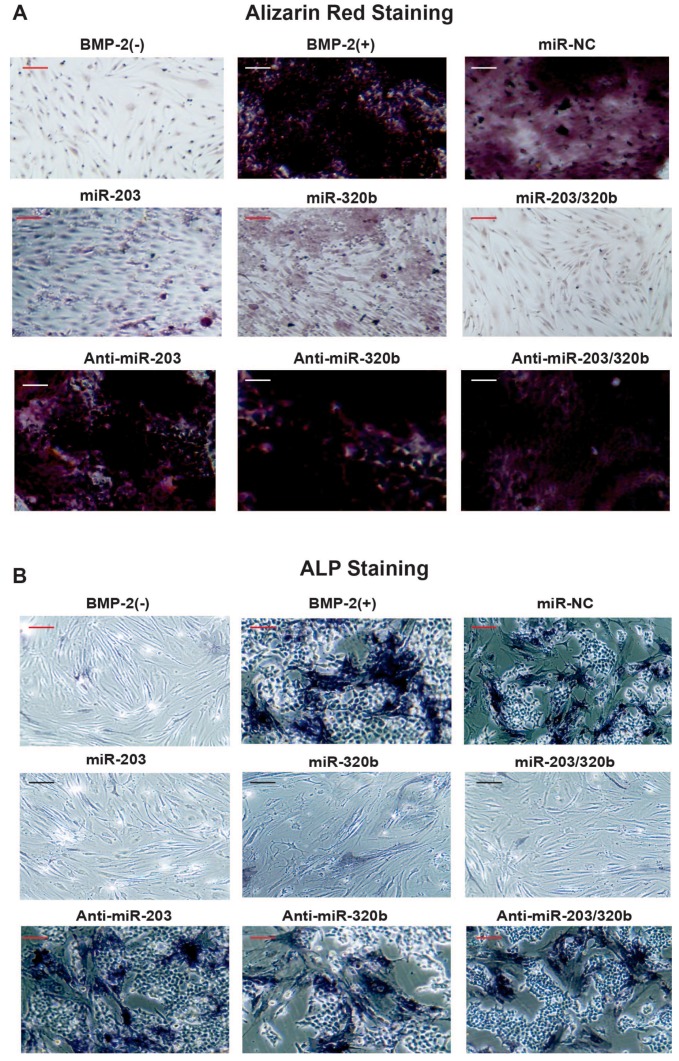
Effects of miR-203 and miR-320b on nodule formation and ALP activity. Human osteoblast cells were cultured and were transfected with miR-203, miR-320b miRs or Anti-miRs and with negative control (NC), and cells were stimulated with BMP-2 for 12 days. The deposition of calcium was detected by: (**A**) Alizarin Red staining; and (**B**) an ALP staining assay. Experiments were performed in triplicate and representative micrographs are shown with scale bar = 200 µm.

To study the impact of miR-203 and miR-320b on BMP-2 stimulated differentiation of human osteoblast, HOBs were transfected with either pre-miR or anti-miR. The osteoblast differentiation marker ALP expression was significantly enhanced with the inhibition of miR-203 and miR-320b after BMP-2 treatment compared to no BMP-2 treatment and NC, and in vitro matrix mineralization was visualized by Alizarin red staining. In contrast, HOBs transfected with pre-miR miR-203 and miR-320b, the ALP activity and matrix mineralization was reduced (Figure 5A,B). Taken together, these results suggest that miR-203 and miR-320b act as negative regulators of osteoblast differentiation.

### 3.5. miR-203 and miR-320b Directly Target Dlx5

**Figure 6:**
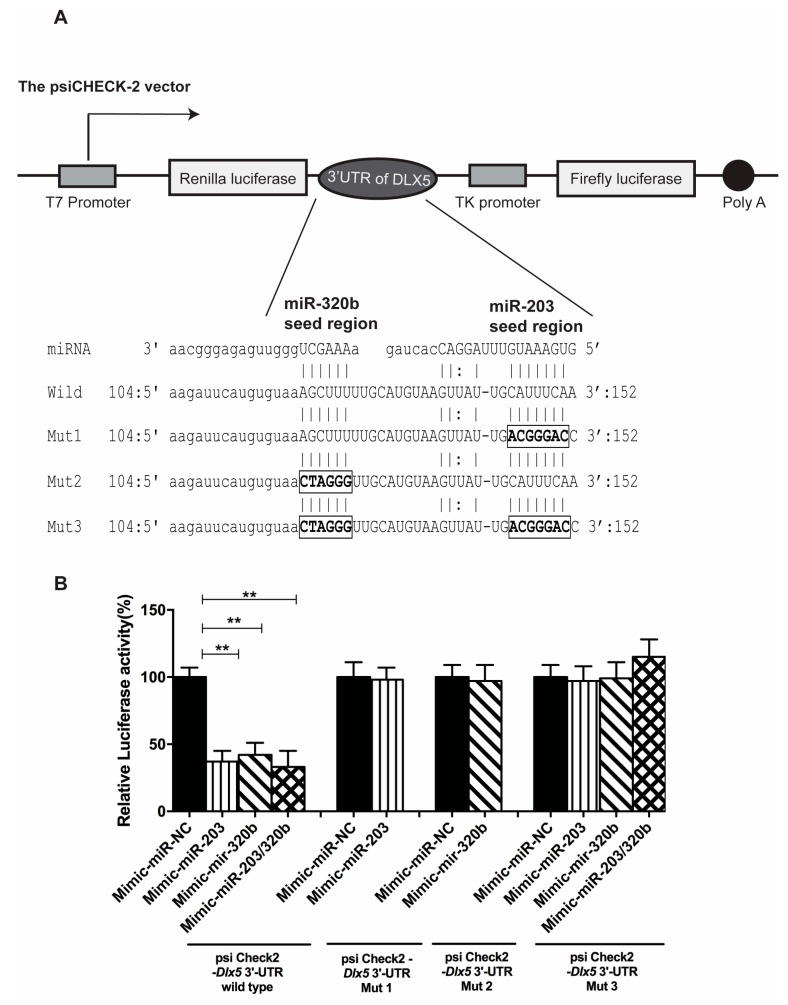
Characterization and functional analyses of the *Dlx5* 3′ UTR. (**A**) Schematic representation of dual luciferase reporter plasmid psiCHECK-2 vector containing the 3′ UTR of *Dlx5* inserted downstream of Renilla luciferase gene. Four types of dual luciferase reporter plasmids were constructed, one wild type and three types of mutations were constructed within the seed region of the miR-203 and miR-320b binding site. (**B**) Effect of miR-203 and miR-320b overexpression on the plasmid containing the 3′ UTR of *Dlx5* with the mutations was analyzed. Firefly and luciferase activity of each construct were measured in cell lysates. The luciferase activity was normalized to the miRNA negative control (NC). All values are represented as mean ± S.D. of three independent experiments. Statistical significance is indicated as and ** *p* < 0.001.

Target prediction algorithms TargetScan, PicTar and miRanda were utilized identifying *Dlx5* as a potential target gene for miR-203 and miR-320b. To determine whether miR-203 and miR-320b directly target *Dlx5*, a *Renilla* luciferase reporter was constructed in the psiCHECK-2 vector containing the 3′ UTR (Untranslated region) of *Dlx5* (wild-type). In addition, three types of mutant luciferase reporter plasmids were constructed, containing mutation in the 3′ UTR of *Dlx5* (Figure 6A). The wild-type and the three mutant plasmids were co-transfected with pre-miR-203 and pre-miR-320b in HOBs and the level of firefly and *Renilla* luciferase activity was measured consecutively. Luciferase activity was suppressed in cells transfected with the wild-type plasmid overexpressed with either miR-203 or miR-320b or both compared to NC. Mutations at the binding site of both the miRNA abolished this decrease in luciferase activity (Figure 6B). In all experiments, the psiCHECK-2 vector constitutively expressing firefly luciferase activity served as a normalization control for transfection efficiency. These results strongly indicate that miR-203 and miR-320b specifically target *Dlx5*.

## 4. Discussion

**Figure 7:**
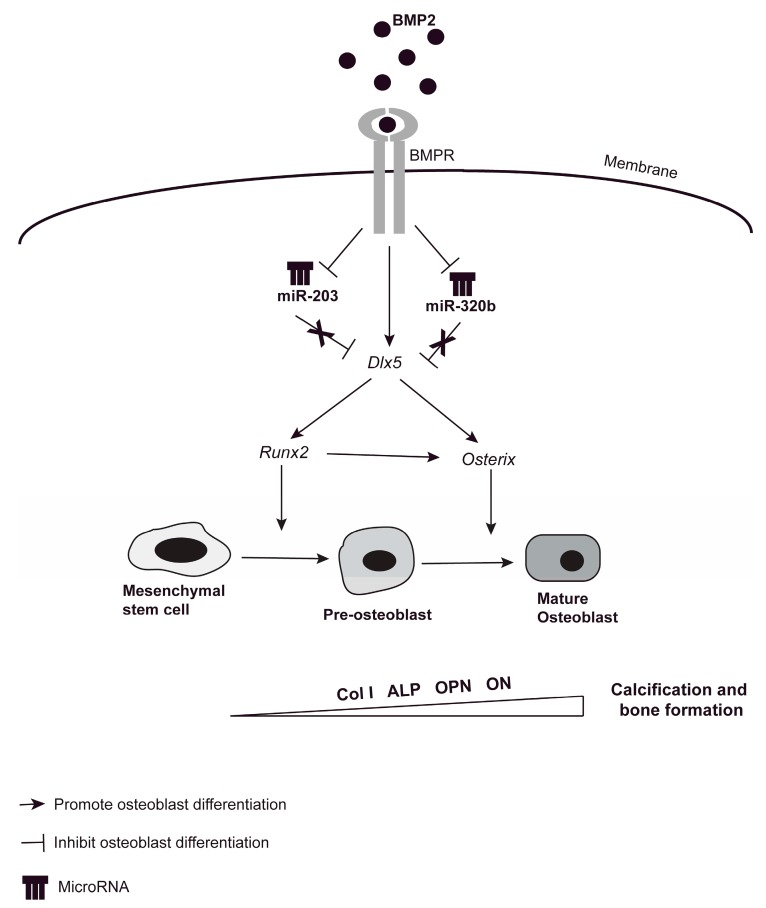
Schematic outline highlighting the complex regulation of BMP-2-induced osteoblastogenesis. BMP-2 binding to the BMP-2 Receptor (BMPR) activates the canonical BMP signaling pathway that regulates osteoblastic differentiation. Our present results suggest that BMP-2 downregulates the expression of miR-203 and miR-320b, in turn upregulating the transcription factor *Dlx5* (Distal-less Homeobox 5). *Dlx5* up regulation activates both the transcription factors *Runx2* and *Osx*, promoting osteoblast differentiation. This in turn results in the upregulation of genes important for bone formation and calcification, e.g., collagen type I (*COL1A1*), alkaline phosphatase (ALP), Osteopontin (OPN) and osteonectin (ON).

BMP-2 is a highly efficient inducer of osteogenesis, regulating osteoblast differentiation by binding to and thereby stimulating the BMP receptors (BMPR) BMPR-I and BMPR-II. The downstream regulators Smad proteins play an important role in relaying BMP signals from receptors to activate the expression of three osteogenic master transcription factors *Dlx5*, *Runx2* and *Osx* in the nucleus [34]. *Runx2* in turn regulates the expression of many osteoblastic genes, e.g., *Col1A1* (Collagen type I AI), alkaline phosphatase (*ALP*), Osteopontin (*OPN*) and osteonectin (*ON*) [35,36] (Figure 7). Many bone regulating miRNAs have been recently identified exerting their effects at different stages of osteoblast differentiation when stimulated with BMP-2. Studies show that Smad proteins may bind to miRNA promoter genes and control their transcription and regulate miRNA biogenesis and processing [37,38,39,40]. Furthermore, during BMP-2 induced osteogenesis, the expressions of many osteomiRs are downregulated.

To our best knowledge, this is the first study that relates miR-203 and miR-320b to *Dlx5* in a BMP-2-induced human osteoblast differentiation. Previous studies have shown that miR-141 and miR-200a regulate BMP-2 mediated osteoblast differentiation by targeting *Dlx5* [22], miR-208 target *Ets1* and regulate BMP-2 induced mouse preosteoblast differentiation [41]. miR-203 has previously been shown to target *Runx2* in osteolytic bone disease [42,43]. Our study was conducted in primary human osteoblast cells, and we used BMP-2 to induce osteoblast differentiation. Based on previous studies conducted in our group [33], we identified miR-203 and miR-320b as miRNAs important during osteoblast differentiation. Our results indicate that miR-203 and miR-320b, at least in part, regulate osteoblast differentiation by downregulating a common target, *Dlx5*. Studies have shown that several miRNAs may bind to their target mRNAs and cooperatively fine-tune the degree of protein translation of a single mRNA [44].The regulation of common target genes in which upstream regulators also have an impact on each other, is reminiscent of feed-forward loops established within a transcription factors-based hierarchy. *Dlx5*. We validated the *Dlx5* as a target of miR-203 and miR-320b using Luciferase assays. BMP-2 downregulates miR-203 and miR-320b in a time dependent manner.

Studies by Ulsamer and Holleville et al [45,46] have shown that *Dlx5* regulates the expression of *Runx2* and *Osx* and their expression is upregulated when induced by BMP-2. To assess the effect of miR-203 and miR-320b on *Dlx5* in regulating osteoblast differentiation, we overexpressed miR-203 and miR-320b and observed that osteoblast differentiation was suppressed by the decrease in the expression of *Dlx5*, *Runx2* and *Osx*. Reciprocally, we also observed that when miR-203 and miR-320b are repressed, this results in an increased expression of the all three transcription factors. Although there was a clear effect by knockdown of miR-203 and miR-320b increasing the levels of Dlx5, Runx2 and Osx by 70%–80%, effects of other modulating factors including other osteogenic miRNAs cannot be ruled out. ALP activity was also diminished and mineral nodule formation was suppressed with miR-203 and miR-320b overexpression. In this paper, we demonstrate for the first time that miRNA 203 and miR-320b regulate *Dlx5* in a human model of BMP-2 induced osteoblast differentiation.

In summary, we have identified two osteoblast related miRNAs that are crucial for regulating osteoblast differentiation. We indicate in our study that miR-203 and miR-320b actively inhibit osteogenesis and BMP-2 addition down regulated the miRNAs and promoted osteoblast differentiation. We observed that miR-203 and miR-320b together mediated repression of the transcription factor *Dlx5*, which is upstream of and activator of *Runx2* and *Osx*, and they are important for the regulation of osteoblast differentiation. In view of these results, it can be suggested that downregulation of miR-203 and miR-320b by BMP-2 augmented the expression of *Dlx5*, hence bone formation. Taken altogether, we describe a novel function of miR-203 and miR-320b in negatively regulating BMP-2-induced osteoblast differentiation by suppressing *Dlx5*, which in turn suppresses the downstream osteogenic master transcription factor *Runx2* and *Osx*. We also show that inhibition of miR-203 and miR-320b can increase osteoblast differentiation. Further studies are needed, investigating the role of miR-203 and miR-320b in modulating bone metabolism in vivo.

## 5. Conclusions

In summary, our study reveals a novel function of miR-203 and miR-320b in negatively regulating BMP-2-induced osteoblast differentiation by suppressing *Dlx5*, a bone-inducing transcription factor, which in turn suppresses the downstream osteogenic master transcription factor *Runx2* and *Osx* and together they suppress osteoblast differentiation. Taken together, we propose that miR-203 and miR-320b suppresses BMP-induced osteogenic differentiation by suppressing *Dlx5* and its downstream signaling.

## Supplementary Materials

Supplementary File 1

## Abbreviations

The following abbreviations are used in this manuscript:

ALP	alkaline phosphatase
BMP-2	Bone morphogenetic protein 2
*Col1A1*	Collagen type I AI
*Dlx5*	Distal-less Homeobox 5
HOBs	primary human osteoblasts
miRNA or miR	microRNA
mRNA	messenger RNA
*Osx*	Osterix
*OPN*	Osteopontin
*ON*	osteonectin
*PPARγ*	Peroxisome proliferator-activated receptor gamma
*Runx2*	Runt-related transcription factor 2
TGFβ	transforming growth factor-β
UTR	Untranslated region

## References

[1] Groeneveld E.H., Burger E.H. (2000). Bone morphogenetic proteins in human bone regeneration. Eur. J. Endocrinol..

[2] Chen G., Deng C., Li Y.P. (2012). TGF-beta and BMP signaling in osteoblast differentiation and bone formation. Int. J. Biol. Sci..

[3] Akiyama S., Katagiri T., Namiki M., Yamaji N., Yamamoto N., Miyama K., Shibuya H., Ueno N., Wozney J.M., Suda T. (1997). Constitutively active BMP type I receptors transduce BMP-2 signals without the ligand in C2C12 myoblasts. Exp. Cell. Res..

[4] Yamamoto N., Akiyama S., Katagiri T., Namiki M., Kurokawa T., Suda T. (1997). Smad1 and smad5 act downstream of intracellular signalings of BMP-2 that inhibits myogenic differentiation and induces osteoblast differentiation in C2C12 myoblasts. Biochem. Biophys. Res. Commun..

[5] Heldin C.H., Miyazono K., Dijke P. (1997). TGF-[beta] signalling from cell membrane to nucleus through SMAD proteins. Nature.

[6] Miyama K., Yamada G., Yamamoto T.S., Takagi C., Miyado K., Sakai M., Ueno N., Shibuya H. (1999). A BMP-inducible gene, dlx5, regulates osteoblast differentiation and mesoderm induction. Dev. Biol..

[7] Luo T., Matsuo-Takasaki M., Lim J.H., Sargent T.D. (2001). Differential regulation of Dlx gene expression by a BMP morphogenetic gradient. Int. J. Dev. Biol..

[8] Lee M.H., Kim Y.J., Kim H.J., Park H.D., Kang A.R., Kyung H.M., Sung J.H., Wozney J.M., Kim H.J., Ryoo H.M. (2003). BMP-2-induced Runx2 expression is mediated by Dlx5, and TGF-beta 1 opposes the BMP-2-induced osteoblast differentiation by suppression of Dlx5 expression. J. Biol. Chem..

[9] Lee K.S., Kim H.J., Li Q.L., Chi X.Z., Ueta C., Komori T., Wozney J.M., Kim E.G., Choi J.Y., Ryoo H.M. (2000). Runx2 is a common target of transforming growth factor beta1 and bone morphogenetic protein 2, and cooperation between Runx2 and Smad5 induces osteoblast-specific gene expression in the pluripotent mesenchymal precursor cell line C2C12. Mol. Cell. Biol..

[10] Nakashima K., Zhou X., Kunkel G., Zhang Z.P., Deng J.M., Behringer R.R., de Crombrugghe B. (2002). The novel zinc finger-containing transcription factor osterix is required for osteoblast differentiation and bone formation. Cell.

[11] Ryoo H.M., Hoffmann H.M., Beumer T., Frenkel B., Towler D.A., Stein G.S., Stein J.L., van Wijnen A.J., Lian J.B. (1997). Stage-specific expression of Dlx-5 during osteoblast differentiation: Involvement in regulation of osteocalcin gene expression. Mol. Endocrinol..

[12] Lee M.H., Kim Y.J., Yoon W.J., Kim J.I., Kim B.G., Hwang Y.S., Wozney J.M., Chi X.Z., Bae S.C., Choi K.Y. (2005). Dlx5 specifically regulates Runx2 type II expression by binding to homeodomain-response elements in the Runx2 distal promoter. J. Biol. Chem..

[13] Lee H.L., Kim Y.J., Yoon W.J., Kim J.I., Kim B.G., Hwang Y.S., Wozney J.M., Chi X.Z., Bae S.C., Choi K.Y. (2013). Dlx5 inhibits adipogenic differentiation through down-regulation of PPAR?. J. Cell. Physiol..

[14] Nishimura R., Hata K., Harris S.E., Ikeda F., Yoneda T. (2002). Core-binding factor alpha 1 (Cbfa1) induces osteoblastic differentiation of C2C12 cells without interactions with Smad1 and Smad5. Bone.

[15] Stefani G., Slack F.J. (2008). Small non-coding RNAs in animal development. Nat. Rev. Mol. Cell. Biol..

[16] Bartel D.P. (2004). MicroRNAs: Genomics, biogenesis, mechanism, and function. Cell.

[17] Doench J.G., Sharp P.A. (2004). Specificity of microRNA target selection in translational repression. Genes Dev..

[18] Zhao X., Xu D., Li Y., Zhang J.Y., Liu T.T., Ji Y.L., Wang J.F., Zhou G.M., Xie X.X. (2014). MicroRNAs regulate bone metabolism. J. Bone Miner. Metab..

[19] Eguchi T., Watanabe K., Hara E.S., Ono M., Kuboki T., Calderwood S.K. (2013). OstemiR: A novel panel of microRNA biomarkers in osteoblastic and osteocytic differentiation from mesencymal stem cells. PLoS ONE.

[20] Hu R., Li H., Liu W., Yang L., Tan F.Y., Luo X.H. (2010). Targeting miRNAs in osteoblast differentiation and bone formation. Exp. Opin. Ther. Targets.

[21] Taipaleenmaki H., Hokland L.B., Chen L., Kauppinen S., Kassem M. (2012). Mechanisms in endocrinology: micro-RNAs: Targets for enhancing osteoblast differentiation and bone formation. Eur. J. Endocrinol..

[22] Itoh T., Nozawa Y., Akao Y. (2009). MicroRNA-141 and -200a are involved in bone morphogenetic protein-2-induced mouse pre-osteoblast differentiation by targeting distal-less homeobox 5. J. Biol. Chem..

[23] Li Z., Hassan M.Q., Volinia S., van Wijnen A.J., Stein J.L., Croce C.M., Lian J.B., Stein G.S. (2008). A microRNA signature for a BMP2-induced osteoblast lineage commitment program. Proc. Natl. Acad. Sci. USA.

[24] Yang L., Cheng P., Chen C., He H.B., Xie G.Q., Zhou H.D., Xie H., Wu X.P., Luo X.H. (2012). miR-93/Sp7 function loop mediates osteoblast mineralization. J. Bone Miner. Res..

[25] Inose H., Ochi H., Kimura A., Fujita K., Xu R., Sato S., Iwasaki M., Sunamura S., Takeuchi Y., Fukumoto S. (2009). A microRNA regulatory mechanism of osteoblast differentiation. Proc. Natl. Acad. Sci. USA.

[26] Grundberg E., Adoue V., Kwan T., Ge B., Duan Q.L., Lam K.C.L., Koka V., Kindmark K., Weiss S.T., Tantisira K. (2011). Global analysis of the impact of environmental perturbation on cis-regulation of gene expression. PLoS Genet..

[27] Grundberg E., Kwan T., Ge B., Duan Q.L., Lam K.C.L., Koka V., Kindmark A., Mallmin H., Dias J., Verlaan D.J. (2009). Population genomics in a disease targeted primary cell model. Genome. Res..

[28] Laxman N., Rubin G.J., Mallmin H., Nilsson O., Pastinen T., Grundberg E., Kindmark A. (2015). Global miRNA expression and correlation with mRNA levels in primary human bone cells. RNA.

[29] Krek A., Grün D., Poy M.N., Wolf R., Rosenberg L., Epstein E.J., MacMenamin P., da Piedade I., Gunsalus K.C., Stoffel M. (2005). Combinatorial microRNA target predictions. Nat. Genet..

[30] Enright A.J., John B., Gaul U., Tuschl T., Sander C., Marks D.S. (2003). MicroRNA targets in *Drosophila*. Genome. Biol..

[31] John B., Enright A.J., Aravin A., Tuschl T., Sander C., Marks D.S. (2004). Human MicroRNA targets. PLoS Biol..

[32] Livak K.J., Schmittgen T.D. (2001). Analysis of relative gene expression data using real-time quantitative PCR and the 2(-Delta Delta C(T)) Method. Methods.

[33] Laxman N., Rubin C.J., Mallmin H., Nilsson O., Tellgren-Roth C., Kindmark A. (2016). Second generation sequencing of microRNA in human bone cells treated with parathyroid hormone or dexamethasone. Bone.

[34] Nohe A., Hassel S., Ehrlich M., Neubauer F., Sebald W., Henis Y.I., Knaus P. (2002). The mode of bone morphogenetic protein (BMP) receptor oligomerization determines different BMP-2 signaling pathways. J. Biol. Chem..

[35] Ducy P., Karsenty G. (1995). Two distinct osteoblast-specific cis-acting elements control expression of a mouse osteocalcin gene. Mol. Cell. Biol..

[36] Javed A., Barnes G.L., Jasanya B.O., Stein J.L., Gerstenfeld L., Lian J.B., Stei G.S. (2001). Runt homology domain transcription factors (Runx, Cbfa, and AML) mediate repression of the bone sialoprotein promoter: Evidence for promoter context-dependent activity of Cbfa proteins. Mol. Cell. Biol..

[37] Blahna M.T., Hata A. (2012). Smad-mediated regulation of microRNA biosynthesis. FEBS Lett..

[38] Davis B.N., Hilyard A.C., Lagna G., Hata A. (2008). SMAD proteins control DROSHA-mediated microRNA maturation. Nature.

[39] Davis B.N., Hilyard A.C., Nguyen P.H., Lagna G., Hata A. (2010). Smad proteins bind a conserved RNA sequence to promote microRNA maturation by Drosha. Mol. Cell.

[40] Butz H., Rácz K., Hunyady L., Patócs A. (2012). Crosstalk between TGF-β signaling and the microRNA machinery. Trends Pharmacol. Sci..

[41] Itoh T., Takeda S., Akao Y. (2010). MicroRNA-208 modulates BMP-2-stimulated mouse preosteoblast differentiation by directly targeting V-ets erythroblastosis virus E26 oncogene homolog 1. J. Biol. Chem..

[42] Taipaleenmäki H., Browne G., Akech J., Zustin J., van Wijnen A.J., Stein J.L., Hesse E., Stein G.S., Lian J.B. (2015). Targeting of Runx2 by miR-135 and miR-203 impairs progression of breast cancer and metastatic bone disease. Cancer Res..

[43] Saini S., Majid S., Yamamura S., Tabatabai L., Suh S.O., Shahryari V., Chen Y., Deng G., Tanaka Y., Dahiya R. (2011). Regulatory Role of mir-203 in prostate cancer progression and metastasis. Clin. Cancer Res..

[44] Bartel D.P., Chen C.Z. (2004). Micromanagers of gene expression: The potentially widespread influence of metazoan microRNAs. Nat. Rev. Genet..

[45] Ulsamer A., Ortuño M.J., Ruiz S., Susperregui A.R.G., Osses N., Rosa J.L., Ventura F. (2008). BMP-2 induces Osterix expression through up-regulation of Dlx5 and its phosphorylation by p38. J. Biol. Chem..

[46] Holleville N., Matéosa S., Bontouxc M., Bollerotd K., Monsoro-Burq A.H. (2007). Dlx5 drives Runx2 expression and osteogenic differentiation in developing cranial suture mesenchyme. Dev. Biol..

