# Supplementary figures and images for: miR-203 and miR-320 Regulate Bone Morphogenetic Protein-2-Induced Osteoblast Differentiation by Targeting Distal-Less Homeobox 5 (Dlx5)
Source: Genes (Basel). 2016 Dec 23;8(1):4. doi: 10.3390/genes8010004 (PMC5294999; doi:10.3390/genes8010004)

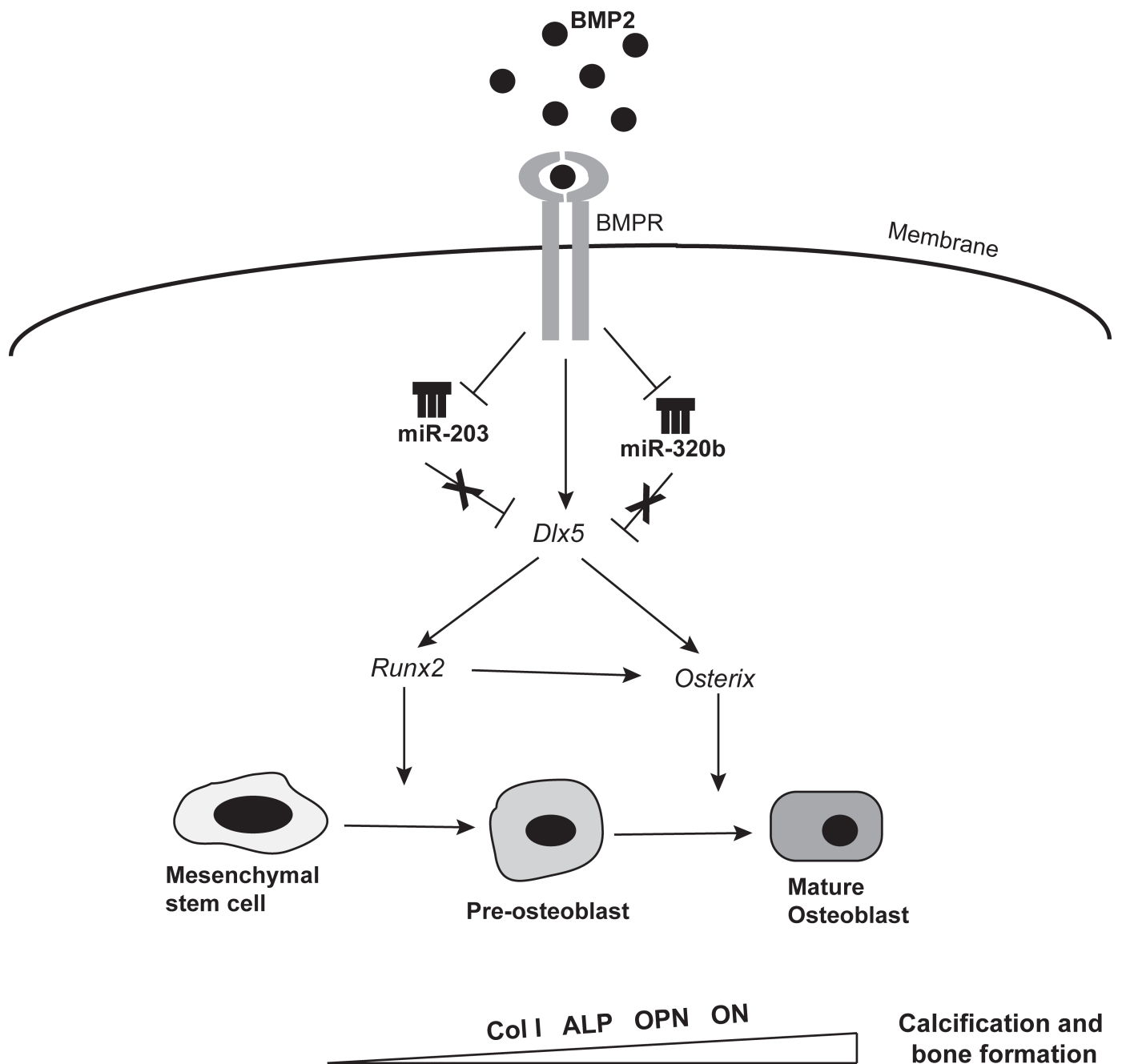

→ Promote osteoblast differentiation

—| Inhibit osteoblast differentiation

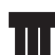 MicroRNA

Supplement: Supplementary File 1 [file genes-08-00004-s001.pdf]
